# Writing knowledge, practices, efficacy, interests, attitudes, and beliefs of deaf education teachers: a randomized controlled trial

**DOI:** 10.3389/fpsyg.2023.1214246

**Published:** 2023-07-07

**Authors:** Kimberly Wolbers, Hannah Dostal, Steve Graham, Lee Branum-Martin, Thomas Allen, Leala Holcomb, Rachel Saulsburry

**Affiliations:** ^1^The University of Tennessee, Knoxville, TN, United States; ^2^University of Connecticut, Storrs, CT, United States; ^3^Arizona State University, Tempe, AZ, United States; ^4^Georgia State University, Atlanta, GA, United States; ^5^Gallaudet University, Washington, DC, United States

**Keywords:** professional development, writing instruction, deaf education, teacher beliefs, efficacy, instructional practices

## Abstract

Writing teachers play an extraordinarily important role in their students’ writing development. Teachers’ motivational beliefs, such as attitudes toward writing, perceptions of their efficacy to teach writing, or preparation to use evidence-based instructional practices, impact their writing instruction, which directly affects the advancement of students’ writing skills. Deaf writers are a subpopulation of writers who may face discriminatory beliefs toward their writing development stemming from ableism, audism, or linguicism. Deaf education teachers may doubt their abilities to teach bilingual/multilingual students or teach deaf students experiencing language deprivation. The current study investigates whether deaf education teachers’ beliefs can be fostered through an intensive one-year professional development (PD) program designed specifically for deaf education teachers. In this randomized controlled trial, we examine the extent to which the participation of deaf education teachers in specialized PD and subsequent writing instruction implementation (*n* = 26) impacts their pedagogical content *knowledge*, use of evidence-based *practices* for teaching writing, *interest*, *attitudes*, *efficacy* in teaching writing, and *epistemological beliefs* about writing compared to teachers in a business as usual condition (*n* = 24). Pre-post regression analyses indicate statistically significant group differences (with the treatment group scoring higher) on all variables except attitude and some epistemological beliefs. We speculate that specialized, sustained PD paired with supported implementation of writing instruction and ongoing teacher reflection are contributing factors to changes in teachers’ motivational beliefs.

## Literature review

Writing is an integral component of people’s daily lives across the globe. It is used for practical purposes, such as creating grocery lists, initiating a petition, or chronicling life experiences. Writing development does not occur innately; rather, it necessitates continuous effort and practice over time. Teachers play a pivotal role in fostering students’ writing development, with instruction often commencing in preschool and extending beyond the twelfth grade. Effective teacher preparation is critical to enhancing the quality of instruction, which subsequently influences student achievement. This study is an investigation into an intensive, sustained professional development (PD) program tailored for teachers of deaf students.

### Theoretical framework

Inclusive of social and cognitive perspectives, the Writer(s)-within-Community model (Graham, 2018, 2023) provides a broad lens for examining influencing factors on how writing is taught. Writing instruction occurs in context-specific environments involving writers, readers, teachers, and collaborators, and each member carries with them unique experiences, abilities, and motivations. Relevant to this study, the Writer(s)-within-Community model can be used to predict and explain the cognitive capabilities and resources that teachers bring to the act of teaching, with a focus on their knowledge and beliefs, as they are retrieved from memory and acted upon in the teaching of writing (see Graham, 2023). In addition, one’s actions are moderated by emotions and personality traits. For example, teachers who hold considerable knowledge about how to teach writing likely hold greater confidence and positivity about their instructional competencies. While no writing community is exactly the same because writing instruction is influenced differently by teachers, a common experience such as a PD program has the potential to impact teachers’ knowledge and beliefs. In the experimental study that follows, we examine the impact that a PD program has on teacher-level variables including knowledge, self-efficacy, and implementation of evidence-based instructional practices. In addition, as teachers make gains in these ways, we inquire into the simultaneous impact a PD program may have on other potentially relevant variables such as teachers’ interest in teaching writing, attitude about writing, and epistemological beliefs.

### Teacher as a factor in writing instruction and learning

Teachers are one of the most influential factors in students’ academic success. Their knowledge and educational preparedness are directly associated with student achievement (Burroughs et al., 2019). With respect to the teaching of writing, a teacher’s cognitive capabilities and resources – such as one’s knowledge of writing instruction, application of evidence-based practices, and efficacy – play a significant role in how writing is taught in the classroom, and these may impact or be impacted by other factors such as interest, attitudes, beliefs, or state/school policies, to name a few. There are a number of cognitive and social factors influencing writing instruction, and one’s preparation to teach writing has potential to positively impact them. A study by Graham et al. (2023) of 143 general and special education teachers of elementary students receiving special education services found that when a teacher holds positive beliefs about their preparation to teach writing, they are likely to provide more favorable reports of their knowledge, attitude toward writing, attitude toward teaching writing, and belief that writing can be developed through effort and process.

Another relevant finding of the Graham et al. (2023) study was that general education teachers held more favorable beliefs than special education teachers. Teachers of deaf students must possess both generalized and specialized knowledge to provide writing instruction that responds to students’ unique and diverse language needs (Dostal et al., 2019). Thus, it is even more critical that PD programs are able to affect deaf education teachers’ outcomes positively. Understanding the influence that a PD program has on teacher variables is crucial, as one’s capacities, resources, and beliefs can either enhance or hinder instructional effectiveness and student outcomes. Researchers and educators can design PD programs that lead to change in teacher factors and foster more effective student learning experiences.

#### Knowledge of writing instruction and use of evidence-based practices

Knowledge of writing instruction refers to teachers’ understanding of the principles, strategies, and techniques required to effectively teach writing to students with varying abilities. Teachers must possess a deep understanding of the writing processes, writing genres, and strategies that support writing development across diverse learners. Research demonstrates that low teacher knowledge is correlated to low student knowledge (Piasta et al., 2009; Binks-Cantrell et al., 2012). In the context of writing instruction, Wijekumar et al. (2019) found that teachers with low knowledge of text structures had difficulties with teaching strategies for engaging with text structures. Alternatively, teachers with solid knowledge of writing instruction are able to describe teaching practices that are grounded in research and have been shown to improve student writing outcomes. In a study by Wolbers et al. (2016) of elementary deaf students, teachers’ knowledge of writing instruction significantly increased after engaging in PD that embedded information about and the application of evidence-based practices. If a teacher has knowledge of evidence-based practices, it increases the likelihood that these practices will be implemented in the classroom.

Evidence-based practices in writing instruction are identified through rigorous or statistical reviews of writing research conducted with diverse subpopulations of students (Graham and Perin, 2007; Graham et al., 2012), and also specifically with deaf and hard of hearing writers (Strassman and Schirmer, 2013). Explicitly teaching strategies for writing processes (e.g., planning, organizing, revising) is one of the most evidenced approaches, producing large, positive effects for low- and high-achieving writers (e.g., De Silva and Graham, 2015), and deaf writers (e.g., Wolbers et al., 2022). Specific to the teaching of elementary students, evidence-based methods include teaching students to engage in the writing process for diverse purposes (Ferretti et al., 2009; Tracy et al., 2009; Wolbers et al., 2015; Dostal and Wolbers, 2016), to become comfortable with handwriting, spelling, sentence construction, typing, and word processing (Graham et al., 2001; Saddler and Graham, 2005; Wolbers et al., 2020), and to participate in a community of writers (Yarrow and Topping, 2001; Troia and Graham, 2002; Wolbers et al., 2022). The amount of time students spend writing each week also plays a crucial role in their writing development. However, a review of the literature that investigated the current state of writing instruction revealed that the majority of teachers were not adequately employing evidence-based practices (Graham, 2019). Many teachers did not spend enough time teaching writing, did not provide adequate opportunities for students to engage in writing, and did not teach writing strategies. Although deaf education teachers report adequate time for teaching writing, they express an underpreparedness with specialized language approaches (Wolbers et al., 2023) known to be effective in writing instruction with bi−/multi-lingual deaf students and those experiencing language deprivation (Wolbers et al., 2018).

#### Efficacy in writing instruction

Teacher efficacy is important in education because it influences instructional practices and student achievement. Bandura (1978) describes efficacy as teachers’ belief in their ability to impact student outcomes with their instruction. Highly efficacious teachers believe they can positively affect student learning, even when students face challenges such as low socioeconomic status or lack of interest and motivation in school (Salgado et al., 2018). Teachers with high efficacy exert more effort, maintain higher expectations, adapt to new methods, persist through obstacles, and are not as critical of students’ struggles (Zee and Koomen, 2016). High teacher efficacy leads to greater job satisfaction and reduced stress (Caprara et al., 2006). A survey of 296 deaf education teachers revealed that they had a high self-efficacy, which was significantly correlated with their years of teaching experience (Garberoglio et al., 2012). Teachers felt more efficacious in the areas of instructional strategies and classroom management than student engagement. The most impactful predictor of teacher self-efficacy was their perception of the efficacy of the educational program.

Specific to writing instruction, teacher efficacy relates to confidence in one’s ability to teach writing and to improve students’ writing outcomes (Brindle et al., 2016). Research indicates that teachers with high efficacy exhibit positive attitudes toward teaching writing and spend more time supporting students’ writing development (De Smedt et al., 2016; Rietdijk et al., 2018). A survey involving 44 elementary deaf education teachers who taught writing found that they had high self-efficacy and somewhat positive attitudes toward writing, along with partial beliefs that writing skills necessitated effort and practice (Graham et al., 2021). This research found that teacher self-efficacy played a significant role in predicting the reported use of efficacious writing instruction practices. As a result of efficacious teachers, students who face challenges in writing respond positively to instruction (Graham and Harris, 2002) with their writing showing growth (Zee and Koomen, 2016; Ekholm et al., 2018). In contrast, a study by Brindle et al. (2016) revealed that many elementary teachers reported low efficacy, indicating a lack of confidence in their ability to teach and enhance students’ writing skills. Variations in teacher efficacy correspond with the Writer(s)-within-Community model, where teachers possess diverse cognitive capabilities and resources that affect instruction and learning.

#### Additional teacher variables and their relationships

Research on additional teacher-related factors such as interest in writing instruction, attitudes toward writing, and epistemological beliefs about writing instruction is minimal (Graham et al., 2022). Interest in writing instruction addresses the level of engagement and enthusiasm teachers have toward teaching writing, and attitudes toward writing encompass teachers’ feelings and perceptions about engaging in the act of writing (Brindle et al., 2016). Epistemological beliefs about writing instruction denote the underlying assumptions teachers have about the nature of writing skills, such as whether skills are innate or learned through practice (Hsiang et al., 2020). The extent to which attitudes and beliefs may interact with or occur simultaneously to more established constructs such as knowledge of writing instruction, use of evidence-based practices, and efficacy in teaching writing remains largely unknown.

There is considerable variability in teachers’ beliefs, interests, attitudes, and writing practices in teaching writing across different countries and grade levels (Troia and Graham, 2002; Brindle et al., 2016; De Smedt et al., 2016; Rietdijk et al., 2018; Graham et al., 2022). Hsiang and Graham (2016) and Hsiang et al. (2018, 2020) conducted a study in China and Taiwan that demonstrated teachers with positive beliefs, interests, and attitudes are more likely to apply evidence-based instructional practices. Graham et al. (2021) and Bañales et al. (2020) surveyed teachers in Norway and Chile, respectively, further reinforcing the evidence that teacher interests, attitudes, and beliefs predicted evidence-based instructional practices. However, not all variables had the same impact on writing instruction across different countries, which aligns with the Writer(s)-within-Community model in the diverse affordances of writing communities such as cognitive resources that exist in each individual and the sociocultural factors influencing their experiences, knowledge, and beliefs (Graham et al., 2023).

### Professional development

The premise of the current study is that teacher variables can be positively transformed through high-quality PD (Bifuh-Ambe, 2013; Cremin and Oliver, 2017), which can lead to improved student writing outcomes (Whyte et al., 2007; McMaster et al., 2020; Wolbers et al., 2022). Intensive, sustained PD initiatives with clearly defined goals are more likely to lead to increased pedagogical content knowledge and teaching effectiveness than one-time workshops (Darling-Hammond and Richardson, 2009; Thames and Ball, 2010). Further, robust PD programs should offer supported application of skills contextualized within authentic classroom experiences (Desimone, 2009; Wilson, 2013), and provide teachers with prompt performance feedback (Leko et al., 2015). Scaffolding and ongoing coaching of specific skills are gradually reduced over time as teachers gain confidence and mastery (Leko et al., 2015). In the context of deaf education, Strategic and Interactive Writing Instruction represents a PD program that aligns with research-established quality indicators, targeting the enhancement of teachers’ knowledge and application of evidence-based practices.

#### Strategic and interactive writing instruction

The Strategic and Interactive Writing Instruction (SIWI) program, incorporating intensive and sustained PD for educators, seeks to address the language and writing needs of deaf and hard of hearing students, taking into consideration their diverse linguistic backgrounds (Wolbers, 2008a,b). SIWI comprises evidence-based strategic and interactive instructional methodologies. Through deliberate, co-constructed writing activities with teachers and peers, students learn to plan, draft, revise, and edit their writing for communication with authentic audiences (Dostal et al., 2015). Successful SIWI implementation necessitates high-quality PD for educators, as altering traditional instructional practices can be challenging. Wolbers et al. (2016) explored the impact of a multi-year PD program on teachers’ knowledge and implementation of SIWI, discovering that 1 year of the PD program positively influenced teachers’ comprehension of SIWI principles and their capacity to incorporate these principles into their instructional approaches. Furthermore, teachers’ knowledge and implementation continued to more advanced levels with a second and third year of SIWI PD. Although not experimental in design, this study represented a vital step in designing an intensive, sustained PD experience that yielded substantial improvements in pedagogical knowledge and application among deaf education teachers. To isolate the effects that the SIWI PD program has on teachers’ knowledge and capacities for teaching writing, however, a randomized controlled trial is needed.

Previous studies have focused on the development of students’ language and writing skills rather than the teachers’ development. Wolbers et al. (2012) investigated the writing outcomes of 29 middle school deaf students after 1 year of SIWI implementation, observing substantial improvements in syntactic complexity, vocabulary, and expanded texts. In a repeated measures study comparing 5 weeks of typical writing instruction to 5 weeks of SIWI instruction, Dostal and Wolbers (2014, 2016) observed that deaf students (*n* = 23) produced significantly longer writing as well as longer and more complex American Sign Language (ASL) samples upon receiving SIWI instruction. Wolbers et al. (2015, 2020) conducted single-case research design studies, revealing positive changes to deaf elementary students’ written language (e.g., increased compound and complex sentences, T-unit counts, and verb variance) and writing skills (e.g., inclusion of persuasive and informative writing traits) once SIWI was provided. In a randomized controlled trial of 79 deaf students in grades 3–5, Wolbers et al. (2022) found that students who received SIWI significantly outperformed their comparison group counterparts on a standardized assessment, the Woodcock-Johnson IV Broad Written Language, thus highlighting the effectiveness of SIWI in bolstering deaf students’ writing and language abilities. The current study represents the first experimental examination of the ways in which SIWI PD and subsequent implementation effect change in the teachers who provide efficacious writing instruction to deaf students.

### The current study

This study aligns with the Writer(s)-within-Community model, which suggests that the contexts in which teachers operate are adaptable based on internal and external forces, thereby influencing their writing instruction. Teachers possess the potential to transform the writing community through their decisions and actions, derived from newly acquired knowledge and skills from PD, collaboration with other teachers, and supported application and coaching. Thus, experimental studies are encouraged to assess the efficacy of PD in enhancing teachers’ capabilities (Graham, 2023).

Writing communities are not predetermined; rather, they exhibit organic variations based on the contributions of all participants, with teachers serving as one significant influential factor. In the current study, we examine the extent to which SIWI PD impacts teacher-level variables. Specifically, we address two research questions:

1. (Main question) To what extent does SIWI PD appear to improve teacher’s knowledge, use of evidence-based practices, and efficacy for teaching writing?
2. (Exploratory question) To what extent does SIWI PD appear to improve teachers’ interest in teaching writing, attitudes toward writing, and epistemological beliefs about writing?

As a result of teacher engagement in SIWI PD and SIWI implementation, we hypothesize a positive shift in the main teacher factors--greater pedagogical knowledge, increased use of evidence-based practices, and higher self-efficacy for teaching writing.

## Methods

This RCT included 50 teachers over two academic years. School partners from across the United States were recruited during the grant application process through email and national conference listservs. School partners provided a letter of support for the project to be included in the grant application. Once funded, teachers at partner schools were given priority enrollment. Enrollment was then opened to all interested teachers who had not previously participated in the PD program. The inclusion criteria for teachers included: (1) agreeing to the randomization process, (2) signing a contamination agreement that they would not share SIWI information or materials with other educators, and (3) providing two to two and a half hours of writing instruction a week to deaf students in grades 3–6. Upon approval of the study by the Institutional Review Board, teacher consent forms were collected, and teachers were randomly assigned to comparison and experimental groups through computer generated randomization. Comparison group teachers proceeded with their planned writing instruction, while experimental group teachers participated in the year-long SIWI PD program and implemented SIWI with their students. After the year of data collection concluded, comparison group teachers received access to the SIWI PD program. Pre- and post-data were collected through surveys and interviews. The effects of treatment were analyzed using the statistical design of pre-post regression analysis.

### Random assignment

In the first year, more teachers were assigned to the comparison group (*n* = 17) than the treatment group (n = 13) to allow for a waitlist control approach in which teachers who serve 1 year in the comparison group could move into the treatment group in the second year. In the second year, there were more teachers in the treatment group (*n* = 13) than in the comparison group (*n* = 7). Eight of these teachers were newly enrolled and randomized teachers. The waitlist control approach prevented student crossover from treatment to comparison group when more than one teacher participated from the same program. It also aided recruitment and retention of school partners. Randomization adhered to the following rules:

1. Teachers with prior SIWI experience were assigned to the treatment group. One teacher had learned about SIWI in a college class, and one teacher had learned about SIWI from a teacher colleague. In both cases, they had a limited understanding of the approach, yet were both assigned to treatment to avoid contamination.
2. Teachers who were not available to attend summer training were assigned to the comparison group. This applied to one teacher.
3. When there were two or more teachers from the same program, they were evenly assigned to groups. Teachers from schools where there were no other participating teachers were assigned to the comparison group in the first year.
4. Teachers who enrolled after randomization were included in the comparison group. This was applied to three teachers.

### Teacher participants

There were 26 teachers in treatment and 24 teachers in the comparison group. Teachers self-reported demographic data. In the treatment group, all reported as female. There were 2 teachers of color (1 black and 1 Asian/pacific islander) and 24 white teachers. A total of 4 teachers self-identified as Deaf and 22 as hearing; of the Deaf teachers, 2 used hearing aids. In the comparison group, there were 23 female participants and 1 male. There was 1 black teacher, while the remainder were white. A total of 4 teachers self-identified as Deaf, and used a hearing aid. There were a total of 4 native users of ASL in each group; all other teachers learned ASL later in life as a second or additional language. An independent t-test was applied to the number of years teachers have used ASL (treatment *M* = 16.67, SD = 10.96; comparison *M* = 17.43, SD 8.88), which was not statistically significantly different by group, *t*(40) = −0.25, *p* = 0.20. In terms of participants’ highest level of education, there were 6 teachers (2 treatment, 4 comparison) with an Ed.S. degree or Master’s degree plus 30 credits, 36 with a Master’s degree (19 treatment, 17 comparison), and 8 with a Bachelor’s degree (5 treatment, 3 comparison). Independent t-tests were conducted to compare years of teaching experience across groups (treatment *M* = 12.58, SD = 10.81; comparison *M* = 13.33, SD = 9.16), and these were comparable*, t*(48) = −2.66, *p* = 0.27.

Just over half of the teachers worked at one of 8 participating schools for the deaf, while just under half taught at one of 12 participating local education agencies with self-contained classrooms or pull out services for deaf students. Teachers largely reported that their school programs adhered to a bilingual or multilingual education philosophy where ASL and English were utilized for instruction (N = 19 treatment; *N* = 19 comparison). Fewer teachers reported a monolingual approach to education using spoken English and/or some signs paired with speech (*N* = 7 treatment; *N* = 6 comparison).

To further characterize similarities and differences between groups, we asked teachers to rate their preparation to teach writing on a 3 point scale. In the treatment group, 3 teachers rated their writing preparation as exceptional, 18 as adequate, and 5 as minimal. In the comparison group, 4 teachers said their preparation was exceptional, 18 adequate, and 2 minimal. At the start of the year, 20 teachers in the treatment group and 18 in the comparison group indicated they were using a writing curriculum, and 6 in each group said they were not. We list the curricula from most frequently mentioned to least frequently: Lucy Calkins’ Writer’s Workshop (8); Framing Your Thoughts (8); McGraw Hill Wonders (7); Houghton Mifflin Harcourt Journeys (6); National Geographic Inside Series (5); Reading A-Z (4); Bilingual Grammar Curriculum (2); Bedrock Literacy Curriculum (2); 6 + 1 Traits of Writing (1); Orton-Gillingham Approach(1).

One teacher from each group withdrew during the academic year--one due to a change in position and the other due to being overcommitted. Both teachers who withdrew reported as white and hearing.

### Measures

Interview and survey data were collected from teachers in both groups at the beginning and end of the school year. Teachers in the treatment group completed pre-data before starting the SIWI PD program.

#### Interview

Levels of Use of the Innovation (LoU) semi-structured interviews were used to examine teachers’ **knowledge** of writing instruction with deaf students. The LoU is a criterion-referenced measure grounded in the Concerns-based Adoption Model (Hall, 1974) with six operationally defined behavioral profiles (Hall, 2013; Hall and Hord, 2020): non-use (0), orientation (1), preparation (2), mechanical use (3), routine use (4a), refinement (4b), integration (5), renewal (6). Profile 3, routine use, indicates a teacher is reporting their thinking about daily instruction in specific contexts, and changes to instruction are teacher-centered. To score at a level 3 or higher, the teachers’ instruction must contain evidence-based practices for (1) teaching strategies for writing process and skills, (2) apprenticing students as writers through supported writing practice and interaction, and (3) providing specialized language instruction for bilingual/ multilingual students and students experiencing language deprivation. Profile 4, refinement and integration, demonstrates that a teacher is moving beyond mechanical instruction to making student-centered changes to instruction informed by evaluation and motivated by improving student outcomes. Renewal, profile 5, indicates that a teacher is flexibly applying the instructional approach with different students, and also collaborating with other educators and family members to further the impact of instruction. Finally, profile 6 suggests that a teacher is refining and innovating based on reflection of their own practice.

The last two authors and one SIWI coach conducted LOU interviews with each SIWI and BAU teacher prior to the start of the academic year (pre-interview). All teachers were interviewed again at the end of the academic year (post-interview). During a 45 min interview, teachers were asked 24 questions designed to elicit information about their knowledge of writing instruction (e.g., What do you see as the strengths and weaknesses of the writing instruction you are implementing with deaf students? Have you made any attempt to address the weaknesses?) and the impact of their teaching (e.g., What do you see as being the effects of the writing instruction you are implementing with deaf students?).

Each teacher’s interview was transcribed and assessed for knowledge of the characteristics, use, and consequences of the instruction. Scores were based on teachers’ expressed knowledge of instructional practice, which was grounded in classroom-based situations and their interactions with diverse learners. The LOU scoring chart (Hall et al., 2006) includes a description of each score as well as seven decision points (existing between each score) that describe what the teacher is doing. The decision points aid the scorer in determining whether they should advance to the next score level. For example, the first decision point between a score of 0 and 1 states: “Takes action to learn more detailed information about writing instruction.” Whereas the descriptions of scores 0 and 1 for knowledge state: “Knows nothing about this or similar innovations or has only very limited general knowledge of efforts to develop innovations in this area.” (0) and “Knows general information about the innovation such as origin, characteristics, and implementation requirements.” (1). The first two authors and the last author scored LoU interviews. They reviewed the scoring protocol as a group and then scored approximately 20% together to calibrate. The remainder of the interviews were scored by at least 2 members. Any differences in scoring were discussed by the 3-member team to achieve consensus.

#### Survey

Four established surveys were compiled into one online survey for teachers. In one section of the survey, there were 15 items from the *Survey of Evidence-based Practices* (Brindle et al., 2016) that related to teachers’ use of **evidence-based practices while teaching** (8 items) **and supporting writing** (7 items). Teachers responded to the items using an 8-point scale (1 never; 8 several times a day). Example items ask teachers to rate how often they “teach students strategies for planning” (teaching) and “provide feedback on students’ writing” (supporting). Internal consistency (Cronbach’s alpha) for teaching items was 𝜶=0.84 at pre survey and 𝜶=0.87 at post survey; supporting writing items were 𝜶=0.79 at pre survey and 𝜶=0.81 at post survey.

Another section of the survey included 9 items from the *Efficacy for Teaching Writing* survey (Graham et al., 2001; Brindle et al., 2016) that addressed teachers’ **efficacy** in teaching writing using a 6-point scale (1 strongly disagree; 6 strongly agree). For example, teachers were asked to rate the following item: “If a student did not remember what I taught in a previous writing lesson, I would know how to increase their retention in the next lesson.” Internal consistency was 𝜶=0.79 at pre survey and 𝜶=0.82 at post survey.

A third section of the survey included items from a subsection of the *Classroom Practices for Writing* survey (Brindle et al., 2016) to measure **interest** in teaching writing and **attitude** toward writing. One six-point Likert type question asked teachers if they like teaching writing, and six questions of the same type asked teachers to rate the degree to which they agree or disagree with statements regarding their attitude toward writing. An example of a question that measured teachers’ attitudes is “I enjoy learning about becoming a better writer.” Internal consistency of attitude survey items was 𝜶=0.81 at pre survey and 𝜶=0.87 at post survey.

The last section of the survey included 25 items related to teacher’s **epistemological beliefs** about writing from Hsiang et al. (2020). Items assessed four dimensions of beliefs: (a) writing development is **innate** or fixed (e.g., some people are born good writers, others are stuck with limited writing capabilities); (b) writing development occurs through **effort** and process (e.g., with practice, one can become a good writer); (c) writing knowledge comes from **experts** and authority figures (e.g., experts know more about teaching writing than I do, so I rely on their judgment); and (d) writing knowledge is **certain** (e.g., if two people score a student’s writing differently, at least one of them must be wrong). Teachers who have attended the SIWI PD may demonstrate to a greater extent that writing development involves learning effort/process, and less that writing development is innate/fixed. Teachers may also demonstrate to a greater extent that writing knowledge comes from authority/experts, and less that writing knowledge is certain knowledge. Items were rated on a 6-point scale (1 strongly disagree; 6 strongly agree). Internal consistency for innate survey items was 𝜶=0.76 at pre survey and 𝜶=0.67 at post survey. It was 𝜶=0.78 and 𝜶=0.82 for effort survey items, 𝜶=0.60 and 𝜶=0.75 for expert items, and 𝜶=0.51 and 𝜶=0.67 for the certainty of writing knowledge items.

### Research design

The independent variable differentiating treatment and comparison groups was the presence of the SIWI program. All teachers regardless of group provided deaf students in grades 3–6 with writing and language instruction for 2 to 2.5 h a week; however, the treatment group teachers were involved in SIWI PD and subsequent implementation of SIWI as their form of writing instruction. Comparison group teachers continued with their typical writing and language instruction during the academic year (business as usual), after which they were provided the SIWI training. The dependent variables examined in this study include (a) knowledge of writing instruction, (b) evidence-based practices related to teaching writing, (c) evidence-based practices related to supporting writing, (d), efficacy in teaching writing, (e) interest in teaching writing, (f) attitude toward writing, (g) innate epistemological beliefs, (h) effort epistemological beliefs, (i) expert epistemological beliefs, and (j) certain epistemological beliefs. A correlation matrix is available in the Supplementary material.

#### SIWI professional development

The overarching goal of the SIWI PD is to develop teachers’ pedagogical and content knowledge through intensive and sustained programming (Darling-Hammond and Richardson, 2009). The SIWI PD engages teachers in simulated and authentic activities paired with ongoing, contextualized feedback for enacting the driving principles--strategic instruction, interactive instruction, and metalinguistic knowledge and linguistic competence.

Teachers in the treatment group began the PD program by attending a week-long summer workshop. The workshop was structured by cycles of learning, application, and feedback. The experience was cumulative, and teachers were expected to integrate information from each new learning cycle with previously applied knowledge until they were exposed to the full SIWI framework. By the conclusion of the week, teachers began planning how to set up SIWI instruction in their classrooms and how to introduce SIWI to their students. After approximately 2 months of implementation of SIWI in their classrooms, teachers came together for a two-day workshop where they analyzed their students’ writing and planned for a transition to a new genre of writing. In addition to the two in-person workshops, teachers received eight one-on-one virtual coaching sessions *via* Zoom to support implementation of SIWI throughout the academic year. With the exception of the spring semester of 2020 when Covid-19 began impacting the operation of schools, teachers also received two site visits from a SIWI coach.

#### SIWI implementation

Teachers implementing SIWI provided recount, information report, and persuasive writing instruction to their students for approximately 18 h across 9 weeks per genre. The major principles of SIWI were used to plan, teach, and reflect on all writing units. Writing instruction included the co-construction of text in a guided and interactive environment with the teacher and students working collaboratively, and embedded opportunities for shared and independent writing. During writing time, the teacher ensured the students were writing for an authentic purpose to a relevant audience, modeled and invited students to use strategies designed to support engagement in the writing process – including genre-specific features and skills – and employed language zone techniques to clarify and expand students’ use and knowledge of language. For more information about SIWI, see *Enactment of SIWI Principles* and the *SIWI Observation and Fidelity Instrument* at siwi.utk.edu.

##### Instructional fidelity

Teachers video recorded their SIWI lessons and shared these with the research team *via* Swivl platform. From the database of recorded instruction, one unit of each genre of writing instruction per teacher was scored for instructional fidelity. A unit began with determining a purpose and audience for writing and concluded with publishing and sharing the writing. On average units ranged between 5 and 8 lessons.

The SIWI instructional fidelity instrument includes 53 items or indicators of instruction that are organized by major SIWI principles: strategic (e.g., text structure associated with the genre of writing is explicitly discussed); interactive (e.g., learning from one another is encouraged through peer interaction); and, metalinguistic/linguistic (e.g., strategies to get to a point of shared understanding are employed in the language zone). See Dostal and Wolbers (2016) for the full instrument. Each item is given a rating of (1) fully implemented, (0.5) partially implemented, or (0) not implemented to reflect the teacher’s level of implementation. Each teacher’s scores were added up, divided by the maximum possible points, and then multiplied by 100 to convert them into percentages.

Twenty-percent of the units were rated by four research team members to ascertain interrater reliability. The intraclass correlation was 0.87. Afterwards, team members discussed and reached full consensus on the final score. The remainder of the units were scored by one research team member.

The instructional fidelity for treatment group teachers’ units ranged from 47 to 90%, averaging 71% for recount writing instruction, 70% for information report instruction, and 73% for persuasive writing instruction (often the last taught genre of the year). These levels of fidelity are consistent with prior SIWI PD research demonstrating that first-year SIWI teachers average approximately 75% fidelity. With continued implementation and participation in the SIWI PD program after the first year, average fidelity is known to increase to above 90% (Wolbers et al., 2016). Nonetheless, prior studies have demonstrated that first-year SIWI teachers significantly impact their students’ writing and language outcomes even while they are learning to implement with greater fidelity (e.g., Wolbers et al., 2015, 2018, 2020; Dostal and Wolbers, 2016).

#### Differences between treatment and comparison group instruction

Teachers in both the treatment and comparison (or BAU) groups provided writing and language instruction for 2–2.5 h weekly, which was inclusive of recount, informative, and persuasive writing genres. BAU teachers continued with their usual instruction while treatment group teachers implemented SIWI. To describe and distinguish the instruction that was provided to students in the comparison and treatment groups, researchers collected information from teachers *via* a 26-item survey at the beginning and end of the academic year. The questions in the survey were of four types that were randomly placed throughout the survey: (a) evidence-based practices for teaching writing (7 items; e.g., teach students to use genre-specific language and domain-specific vocabulary in their writing); (b) evidence-based practices for supporting writing (8 items; e.g., have students work together to plan, draft, revise, and edit a paper); (c) deaf education practices drawn from the literature (Strassman and Schirmer, 2013; Mayer and Trezek, 2015) and in alignment with SIWI implementation (7 items; e.g., collaboratively problem solve and make decisions about writing with students), and (d) widely used practices in deaf education that are not aligned with SIWI (4 items reverse scored; e.g., have students write a first draft and then a second or final draft). For each item, teachers rated how often they implemented a specific practice on an 8-point likert scale (1 = never, 2 = several times a year, 3 = monthly, 4 = several times a month, 5 = weekly, 6 = several times a week, 7 = daily, 8 = several times a day).

A two sample t-test was performed at the beginning of the academic year (prior to the treatment group receiving SIWI PD) to compare the teachers’ writing instruction practices. There was not a significant difference in instructional practices between the treatment group (*M* = 4.16, SD = 0.80) and the BAU group (*M* = 4.44, SD = 0.54); *t*(48) = −1.45, *p* = 0.15. At the end of the academic year, however, the same independent samples t-test was performed, and significant differences were found between the treatment (*M* = 5.06, SD = 0.68) and BAU (*M* = 4.19, SD = 0.68) groups; *t*(46) = 4.43, *p* < 0.001. As demonstrated through teacher responses to the survey, the treatment group teachers displayed significant increases from pre to post in the frequency with which they engaged in evidence-based writing instruction; these increases were not observed in the BAU group. Changes among SIWI teachers were reflected in strategic instruction (e.g., teaching students strategies for planning, writing paragraphs, revising/editing, and self-regulating the writing process), interactive instruction (e.g., having students work together to plan, draft, revise and edit a paper), and metalinguistic/linguistic instruction (e.g., teaching the differences between ASL and English grammars). For example, during a post LoU interview, one SIWI teacher reflected on the interactive nature of her instruction by reporting that she “saw students start to recognize what they were good at with writing, and that sort of development happened through the [classroom] community as [they] wrote.” Another teacher reflected on how her explicit attention to language during writing instruction allowed for, “a connection between language in print and with expressive ideas and [students] communicating with each other.” There were also increases in genre-specific instruction (e.g., teaching students how different genres are structured), authentic purposes for writing (e.g., having students publish their writing), and using classroom writing data to guide writing instruction. The treatment group also showed a reduction in the frequency with which they used practices not in alignment with SIWI (e.g., teaching grammar using a grammar curriculum or structured approach); whereas, the BAU group showed an increase in the frequency of practices misaligned with SIWI (e.g., editing students’ drafts for them, focusing primarily on grammar instruction). For example, one BAU teacher shared that she requires her students to “use a sentence checklist to make sure each sentence has all of the necessary components” while expecting “students [to] practice implementing the sentence structure, or another specific grammatical component, exemplified in the [teacher’s] model.”

### Data analysis

All teacher outcomes were analyzed with a pre-post regression of the general form:


Posttest=Intercept+Pretest+SIWI+e.

where *Intercept* is the model-predicted outcome (*Posttest*) at the mean of the *Pretest* (mean-centered), *SIWI* is a dummy variable for a teacher in the treatment program, and *e* is random error. All models were fit in SAS PROC MIXED (Littell et al., 2006) so that the residual variance could be used to compute the model-based effect size for the treatment effect (g; Hedges, 2007).

## Results

The main research question of this study was: To what extent does participation in SIWI PD and subsequent implementation impact teachers’ knowledge of writing instruction, use of evidence-based practices for teaching and supporting writing, and efficacy in teaching writing compared to those in a BAU condition? The descriptive statistics for the main teacher outcomes are presented in Table 1 at two time points (beginning and end of the academic year), and the results of the pre-post regression model are presented in Table 2. Three types of estimates are provided in Table 2: fixed effects, random effects, and effect sizes. The fixed effects represent the results of the pre-post regression equation, where intercept represents the model-predicted year-end score for teachers in the control group, pretest is the effect of teachers’ fall score (grand mean centered), and SIWI is the change expected in teachers from baseline to the end of the year (i.e., the treatment effect). The random effect represents the residual variance (and SD), or error. Last, an effect size, Hedges’ *g*, is provided for each variable to demonstrate the SIWI treatment effect (as the treatment effect divided by the residual SD; Hedges, 2007).

**Table 1:** Pre and post outcomes for main research questions.

		Pretest			Posttest		
Outcome	Group	*N*	Mean	*SD*	*N*	Mean	*SD*
Knowledge	BAU	24	0.08	0.28	23	0.09	0.29
	SIWI	26	0.23	0.51	25	3.82	0.43
*Evidence-based practices*							
Teaching writing	BAU	24	4.28	0.94	23	3.97	1.07
	SIWI	26	3.82	1.20	25	5.09	0.99
Supporting writing	BAU	24	4.64	0.59	23	4.29	0.88
	SIWI	26	4.38	1.08	25	5.02	0.92
Efficacy	BAU	24	4.49	0.58	23	4.25	0.62
	SIWI	26	4.34	0.78	25	4.77	0.63

Knowledge represents the mean behavioral profile from 0 to 6, non-use to renewal. Evidence-based practices is the mean score on an 8-point scale (1–8), ranging from never to several times a day. The efficacy score represents the mean of a 6-point scale (1–6), from strongly disagree to strongly agree.

**Table 2:** Model results for pre-post main outcomes.

Effect	Knowledge		Teaching writing		Supporting writing		Efficacy	
Fixed	Est.	SE	Est.	SE	Est.	SE	Est.	SE
Intercept	0.11	0.07	3.89	0.21	4.21	0.17	4.21	0.11
Pretest	0.29	0.12	0.25	0.14	0.45	0.14	0.52	0.11
SIWI	3.69*	0.10	1.27*	0.30	0.90*	0.24	0.59*	0.15
Random	Var.	SD	Var.	SD	Var.	SD	Var.	SD
Residual	0.12	0.35	1.01	1.00	0.68	0.82	0.26	0.51
Effect Size	10.48		1.26		1.08		1.15	

Est., estimate; Var, variance; SE, standard error. *p < 0.05 for fixed effects. Effect sizes are Hedges *g*: the treatment effect divided by the residual SD (Hedges, 2007).

All four main teacher outcomes had statistically significant treatment effects with large effect sizes, suggesting SIWI teachers made considerable gains in knowledge, evidence-based practices, and efficacy for teaching writing that were not observed in BAU teachers. It should be noted that the extremely large effect size on knowledge was due to minimal variance accompanied by no gain in the control group.

In addition, we asked the following exploratory research question: To what extent does participation in SIWI PD and subsequent implementation impact teachers’ interest in teaching writing, attitudes toward writing, and epistemological beliefs? The means and standard deviations for these teacher outcomes at the beginning and end of the year are presented in Table 3. The fixed and random effects and effect sizes of the pre-post regression model are presented in Table 4; these are organized similarly to the results of the main research question.

**Table 3:** Pre and post outcomes for exploratory research questions.

		Pretest			Posttest		
Outcome	Group	*N*	Mean	*SD*	*N*	Mean	*SD*
Interest	BAU	24	4.58	0.97	23	4.43	1.20
	SIWI	26	4.46	1.14	25	5.24	0.72
Attitude	BAU	23	4.52	1.01	23	4.36	0.92
	SIWI	26	4.31	0.90	24	4.37	1.03
*Epistemological Beliefs*							
Effort	BAU	24	4.70	0.72	23	4.60	0.68
	SIWI	26	4.49	0.76	25	4.84	0.68
Innate	BAU	24	2.24	0.84	23	2.18	0.63
	SIWI	26	2.12	0.59	25	2.37	0.67
Expert	BAU	24	3.74	0.65	23	3.48	0.55
	SIWI	26	3.33	0.61	25	3.53	0.97
Certain	BAU	24	3.07	0.54	23	2.87	0.59
	SIWI	26	2.76	0.60	25	2.79	0.64

Outcomes represents the mean of a 6-point scale (1–6), from strongly disagree to strongly agree.

**Table 4:** Model results for pre-post exploratory outcomes.

Effect	Interest		Attitude		Beliefs (Effort)		Beliefs (Innate)		Beliefs (Expert)		Beliefs (Certain)	
Fixed	Est.	SE	Est.	SE	Est.	SE	Est.	SE	Est.	SE	Est.	SE
Intercept	4.43	0.17	4.30	0.12	4.47	0.11	2.12	0.10	3.33	0.15	2.76	0.12
Pretest	0.54	0.11	0.89	0.09	0.67	0.10	0.61	0.10	0.60	0.16	0.53	0.14
SIWI	0.83*	0.23	0.25	0.16	0.44*	0.15	0.28	0.14	0.31	0.22	0.11	0.17
Random	Var.	SD	Var.	SD	Var.	SD	Var.	SD	Var.	SD	Var.	SD
Residual	0.65	0.81	0.30	0.55	0.24	0.49	0.23	0.48	0.50	0.71	0.30	0.55
Effect size	1.03		0.46		0.90		0.58		0.44		0.20	

Est., estimate; Var, variance; SE, standard error. *p < 0.05 for fixed effects. Effect sizes are Hedges *g*: the treatment effect divided by the residual SD (Hedges, 2007).

Teachers who were involved in the SIWI PD experienced statistically significant growth in their interest in teaching writing compared to BAU teachers, and the magnitude of the experimental effect was large. Regarding attitudes toward writing, there was a moderate effect but not a statistically significant difference between groups. By the end of the year, SIWI teachers more strongly agreed that writing develops through effort and process. Group differences were significant and accompanied by a large effect size. Other epistemological beliefs (i.e., innate, expert, certain) did not show notable differences between groups.

## Discussion

This randomized-controlled trial used interviews and surveys to measure the impact of the SIWI PD program on deaf education teachers’ knowledge of writing instruction, use of evidence-based practices (EBPs) for teaching writing, and efficacy in teaching writing--all of which are teacher-related factors of the Writer(s)-within-Community model that influence how writing is taught (Graham, 2018, 2023). Exploratory analyses were additionally conducted for potentially interrelated teacher-level variables including interest in teaching writing, attitudes toward writing, and epistemological beliefs. The results demonstrated the SIWI PD program to have a statistically significant impact and a large effect on all main research variables and two exploratory variables. Significant differences were not identified between groups pertaining to attitudes toward writing and some epistemological belief components. In this section, we discuss the implications of these findings for deaf education and teacher education, shedding light on the importance of high-quality PD programs.

Our findings demonstrate an increase in SIWI teachers’ use of EBPs for teaching and supporting writing, which are practices documented in the literature and in alignment with SIWI practices. Prior to SIWI PD, teachers reported using common practices found within deaf education that are not evidence-based nor aligned with SIWI, such as having students write a first draft and then a second or final draft rather than engaging them in recursive writing and revising processes. Following the SIWI PD, SIWI teachers reported decreasing their use of practices unaligned with SIWI while BAU teachers maintained their use of such practices. Given that teachers in this study were randomized into SIWI or BAU groups, this finding demonstrates that the SIWI PD was effective at increasing teachers’ use of EBP and decreasing other instructional practices that are not established as effective practices.

The features of the SIWI PD align with widely recognized aspects of effective PD, such as active learning, collaboration, expert support, feedback, reflection, and sustained learning (Darling-Hammond et al., 2017). Throughout a year of SIWI implementation, teachers had ongoing conversations with SIWI coaches about their implementation of the instructional principles, with a focus on integrating and increasing the strategies that support student growth. The approach to coaching as a part of a PD program is key, with such strategic planning being more effective than evaluating instructional fidelity (Kennedy, 2016). During the SIWI PD, teachers were encouraged to use the SIWI instructional fidelity instrument to reflect on their pedagogical practices and set instructional objectives for themselves. These goals, taken along with the teachers’ immediate needs for addressing what is currently transpiring in the classroom, provided the direction of the coaching conversations and collaborative planning. The potential for responsive PD to influence teacher change is well documented (e.g., Kennedy, 2016; Darling-Hammond et al., 2017), and in this study, the change was substantial, including teachers integrating and increasing their use of a range of EBPs (see Graham et al., 2012). Among the EBPs for teaching and supporting writing are providing time for students to write daily, teaching them to engage in the writing process with authentic purpose and audience, establishing specific writing goals, supporting their development in constructing sentences, teaching revising strategies, creating a motivated community of writers (Rogers and Graham, 2008; Graham et al., 2012), and teaching the differences between ASL and English grammar (Andrews and Rusher, 2010). This highlights the importance of PD that is embedded and responsive to individual teachers’ use of practices.

Importantly, interviews with teachers demonstrated that SIWI teachers are able to articulate their knowledge of effective writing instruction while providing evidence about how they teach and support student writing. For example, BAU teachers at the beginning and end of the year and SIWI teachers prior to PD reported that they modeled writing skills and then allocated instructional time for their students to apply the skills during independent writing. One teacher shared: “I show my own writing model on Monday morning. Then, students practice implementing the sentence structure, or another specific grammatical component, exemplified in the model.” However, after teachers attended the SIWI PD, they reported a shift in their instructional practice to include modeling for both genre traits and grammar or conventions during the collaborative and supported construction of authentic texts. A teacher noted that through collaborative writing she “saw students start to recognize what they were good at with writing and [their] development [of writing] through the community as we wrote.” As teachers’ instructional practices integrated EBPs, they more frequently shared about improvements they observed in their student’s independent writing.

Reported knowledge of EBPs for writing instruction and use of these practices increased among treatment group teachers in this study, along with beliefs in their abilities to carry out such practices. This aligns with literature that highlights the importance of building pedagogical content knowledge (e.g., Thames and Ball, 2010; Fauth et al., 2019) as well as the importance of teachers’ sense of efficacy (e.g., Tschannen-Moran et al., 1998; Zee and Koomen, 2016). As written by Lauermann and ten Hagen (2021, p. 279):

“Teachers are unlikely to engage in activities that they believe exceed their capabilities and may give up on valued goals if they view these goals as unattainable. Furthermore, teachers’ competence, beliefs, and especially their sense of self-efficacy, have been consistently linked to their long-term commitment to the profession”.

In this way, self-efficacy may be viewed as a key outcome which drives the use of EBPs and creates positive cycles of instruction. As illustrated in the Writer(s)-within-Community model, we understand variables as reciprocally related, whereby the use of EBPs are influenced by and are influencing teachers’ knowledge and efficacy. Given the low efficacy reported by teachers across the SIWI and BAU groups at the start of the study, we are particularly interested in capitalizing on the positive relationship between increased efficacy and use of EBPs. Previous research findings indicate a connection between teachers and their students’ achievement; for example, student achievement and motivation is positively impacted by increased teacher efficacy (Caprara et al., 2006; Barni et al., 2019). Thus, drawing upon research to design quality PD programs for teachers is essential to improving teaching practices in ways that are meaningful to students’ learning. The potential implication of having increased teacher efficacy is an increased belief in students’ competence and capacity as learners.

Findings from this study support the idea that the design of teacher learning should be symmetrical with principles for student learning (Watkins et al., 2018). That is, effective principles of learning designed for students can and should be applied to PD designed for teachers. This is particularly true when it comes to the role that active learning and reflection play in propelling a learner’s practices and knowledge. Such principles of learning are enacted effectively in PD programs through engagement of teachers in analyzing their current teaching approaches in comparison to standards that guide effective professional practice, and by having teachers identify goal areas for their practice and then testing these new teaching practices (Ingvarson et al., 2005). Further, scaffolded learning opportunities that are important to students are equally important to teachers; modeling and providing feedback during initial training and during follow up training sessions are associated with larger magnitudes of effects on teacher practices (Brock et al., 2017). Similar to how thematic units can connect various subject matter and skills that students are learning around one coherent theme, PD programs that are coherent – or connected to teachers’ prior experiences, use of standards and assessments, or professional conversations – are more likely to positively influence changes in practice (Garet et al., 2001). Because SIWI is not a scripted curriculum but rather a comprehensive framework for writing instruction that is composed of driving principles that teachers enact in response to their students’ needs, it is possible for teachers to integrate other curricula, programs, or graphic organizers during enactment. Several coaching conversations with teachers provided support on how to, for example, align their reading curriculum with SIWI, or how to draw on the social studies or science curriculum during writing instruction, or how to embed other literacy programs such as Thinking Maps or Framing Your Thoughts within SIWI rather than teaching these programs separately. The active engagement of teachers along with the scaffolded, coherent, and individualized support provided as part of SIWI PD actualizes evidence-based features of effective PD (Van Driel and Berry, 2012; Kennedy, 2016). These features of learning are symmetrical across students and adults, which reflects the complex nature of learning new knowledge and applying new skills.

### Exploratory teacher variables

In addition to the main variables related to our research questions, we examined six exploratory variables that may illuminate additional teacher-level factors impacted by SIWI PD. The statistically significant increase in teachers’ interest in teaching students writing is accompanied by their changes in reported efficacy and use of EBPs. This suggests that interest grows as competence develops, which can promote a positive cycle of improvement. It is also important for considering the potential trajectory of interest change over time when initiating and supporting teachers’ use of EBPs in writing instruction.

Interestingly, teachers’ own attitudes toward writing did not change significantly across the study, which demonstrates that interest in teaching writing and personal engagement with writing may be independent of one another. A systematic review on teachers’ attitudes toward writing revealed that most teachers, irrespective of their teaching experience, possessed negative perceptions about themselves as writers (Cremin and Oliver, 2017). Most teachers, and even literacy specialists, exhibited mixed attitudes toward writing, with most identifying as avid or passionate readers, rather than writers. Nevertheless, some of these teachers, despite lacking a passion for writing, remained committed and interested in fostering a love for writing in their students (Draper et al., 2000). Brooks (2007) also discovered that effective writing teachers did not write frequently, suggesting that this particular variable on attitudes toward writing may not be crucial for effective instruction when other variables are factored in. However, a few studies observed that teachers’ lack of writer identity negatively impacts their willingness to model the composition process from the perspective of the writer, which in turn, may reduce the quality of writing instruction (Street, 2003; Cremin and Oliver, 2017). Since the SIWI PD program did not enhance teachers’ attitudes toward writing but improved other key domains for effective writing instruction, questions arise about the significance of this particular variable in teaching contexts. It is possible that, much like some professional sports coaches who never played the sport themselves but excel in developing their players, writing teachers may not enjoy writing themselves but can still provide effective writing instruction.

In addition to interest and attitudes related to writing, we examined teachers’ epistemological beliefs about writing. Teachers in the SIWI group reported a change in their belief that one can become a stronger writer with effort and practice. This belief in the malleability in writing proficiency is likely to have a significant impact on the approach to writing instruction and engagement with students during writing activities (Graham, 2023). Given the historically low student performance on average in writing, this belief is important not only for setting and communicating appropriately high expectations for deaf students, but also for a teacher’s belief that their efforts will make a difference. However, for three other belief variables, teachers’ beliefs did not transform after a year of SIWI PD. They demonstrated similar beliefs to BAU teachers about whether writing skills are fixed, writing knowledge comes from authority figures, and writing knowledge is certain. A systematic review of research in the larger literature documented mixed outcomes in teacher beliefs about writing development (Cremin and Oliver, 2017). Some teachers perceived writing ability as fixed, while others believed it could be improved through instruction and practice. Several studies have found that teachers’ beliefs often conflicted with their actual teaching practices, and teachers struggled to reconcile these contradictions (McKinney and Giorgis, 2009; Whitney, 2009; Cremin and Baker, 2010). The findings in our study did not diverge from the literature on these belief variables.

### Limitations

There were four dimensions of epistemological beliefs that were examined in this study--(1) the belief that writing develops through effort and process; (2) the belief that writing development is innate or fixed; (3) the belief that writing knowledge comes from experts; and (4) the belief that writing knowledge is certain knowledge. Teachers in the SIWI group showed an increase from beginning to the end of the year in their belief that writing develops through effort and process; this is a change that was not seen in the BAU teachers. The other dimensions of epistemological beliefs did not demonstrate statistically significant group differences. A potential limitation surrounding these data was the low internal consistency among survey items. Internal consistency for the belief that writing develops through effort was the only dimension with acceptable internal consistency at both pre and post (𝜶﹥0.7). Other dimensions were in the poor to questionable range for internal consistency of survey items (0.5–0.7). There was greater variability in teachers’ scores on the clustered items of these three dimensions, indicating conflicting responses (some high, some low) across items. Therefore, we hypothesize that internal consistency of survey items for the epistemological beliefs clusters impacted findings in these areas. In future research, we propose a larger sample of participants to conduct a factor analysis.

Lastly, we were unable to collect video observations from BAU teachers due to the scale and scope of this study. The inherent risk with self-reported data of instructional practices is the potential for teachers to overstate the elements they implement and the extent to which they apply these elements in their instruction. Despite this risk, our study revealed the BAU teachers reported significant differences in teaching methodologies when compared to SIWI teachers. Although the absence of video observations from BAU teachers could be perceived as a limitation, we believe it did not significantly affect the validity of our results.

### Future directions

The full SIWI PD program is provided to teachers over 3 years. With each year of participation in the program, teachers’ implementation fidelity, and knowledge of writing instruction increases (Wolbers et al., 2016). During the last year of the program, teachers have demonstrated the highest adherence to SIWI instructional principles (mid-90’s), which is in stark contrast to the instructional fidelity levels of first year teachers, as shown in this study (70’s). Future research exploring teachers’ knowledge, efficacy, and use of EBPs across multiple years of participation in the SIWI PD program as their instructional fidelity increases could bolster current findings about the program. In response to a reviewer’s suggestion, we conducted a dosage analysis (not reported here) using the overall fidelity measure. The results of the dosage analysis showed statistically significant, large effects, even larger than those reported here. We report the standard, group-based analysis as yielding conservative, more realistic effects (i.e., with less-than-perfect fidelity).

Additionally, future studies could be extended to explore the ways in which differential levels of instructional fidelity and teacher-related variables impact student writing outcomes. While prior experimental studies evidence that first year SIWI teachers with comparatively low instructional fidelity percentages still have a statistically significant impact on students’ writing outcomes compared to those in a BAU group (Wolbers et al., 2018, 2022), we are interested in whether there is a larger impact on students’ outcomes when receiving instruction from a master SIWI teacher. Lastly, the current study explored teachers’ personal attitudes toward writing, such as if they enjoyed learning about becoming a better writer. These attitudes did not demonstrate significant changes. However, future studies could explore whether SIWI PD leads to a change in teachers’ attitudes about writing instruction.

## Conclusion

Teachers play an extraordinarily important role in writing instruction; in fact, they are central to its success. Further, teachers of deaf students must possess additional specialized knowledge to provide writing instruction that responds to students’ varying language needs. Greater teacher pedagogical knowledge, use of evidence-based practices, and higher self-efficacy in writing have a direct impact on how teachers approach writing instruction in the classroom. In this randomized controlled trial of 50 deaf education teachers, those receiving 1 year of SIWI PD and ongoing coaching increased their knowledge and implementation of evidence-based practices in writing instruction and positively reframed their beliefs about their ability to teach writing as well as their students’ abilities to improve their writing. In previous research, these factors have been shown to have a direct, positive effect on students’ writing outcomes. This underscores the importance of evidence-based PD programs for teachers of writing. The SIWI PD is a sustained, coherent program that pairs active learning with supported implementation of writing instruction and ongoing teacher reflection, which leads to statistically significant changes in deaf education teachers’ knowledge and use of empirically supported writing practices.

## Data availability statement

The raw data supporting the conclusions of this article will be made available by the authors, without undue reservation.

## Ethics statement

The studies involving human participants were reviewed and approved by Institutional Review Board, University of Tennessee with authorization agreements filed with University of Connecticut, Georgia State University, and Arizona State University. The participants provided their written informed consent to participate in this study.

## Author contributions

KW, HD, SG, LB-M, and TA contributed to conception and design of the study. RS coordinated the data processing and scoring. KW developed the database with assistance from RS. LB-M performed the statistical analysis. KW, HD, and LH wrote the first draft of the manuscript. All authors contributed to manuscript revisions.

## Funding

The research reported here was supported by the Institute of Education Sciences, U.S. Department of Education, through Grant R324A170086 to the University of Tennessee. The opinions expressed are those of the authors and do not represent the views of the Institute or the U.S. Department of Education.

## Conflict of interest

The authors declare that the research was conducted in the absence of any commercial or financial relationships that could be construed as a potential conflict of interest.

## Publisher’s note

All claims expressed in this article are solely those of the authors and do not necessarily represent those of their affiliated organizations, or those of the publisher, the editors and the reviewers. Any product that may be evaluated in this article, or claim that may be made by its manufacturer, is not guaranteed or endorsed by the publisher.

## References

[ref1] Andrews J. F. Rusher M. (2010). Codeswitching techniques: evidence-based instructional practices for the ASL/English bilingual classroom. Am. Ann. Deaf 155, 407–424. doi: 10.1353/aad.2010.0036, PMID: 21305977

[ref2] Bañales G. Ahumada S. Graham S. Puente A. Guajardo M. Muñoz I. (2020). Teaching writing in grades 4–6 in urban schools in Chile: a national survey. Read. Writ. 33, 2661–2696. doi: 10.1007/s11145-020-10055-z

[ref3] Bandura A. (1978). Self-efficacy: toward a unifying theory of behavioral change. Adv. Behav. Res. Ther. 1, 139–161. doi: 10.1016/0146-6402(78)90002-4 847061

[ref4] Barni D. Danioni F. Benevene P. (2019). Teachers’ self-efficacy: the role of personal values and motivations for teaching. Front. Psychol. 10:e01645. doi: 10.3389/fpsyg.2019.01645, PMID: 31354604 PMC6640028

[ref5] Bifuh-Ambe E. (2013). Developing successful writing teachers: outcomes of professional development exploring teachers’ perceptions of themselves as writers and writing teachers and their students’ attitudes and abilities to write across the curriculum. Engl. Teach. Pract. Crit. 12, 137–156.

[ref6] Binks-Cantrell E. Washburn E. K. Joshi R. M. Hougen M. (2012). Peter effect in the preparation of reading teachers. Sci. Stud. Read. 16, 526–536. doi: 10.1080/10888438.2011.601434

[ref7] Brindle M. Graham S. Harris K. Hebert M. (2016). Third and fourth grade teacher’s classroom practices in writing: a national survey. Read. Writ. 29, 929–954. doi: 10.1007/s11145-015-9604-x

[ref8] Brock M. E. Cannella-Malone H. I. Seaman R. L. Andzik N. R. Schaefer J. M. Page E. J.. (2017). Findings across practitioner training studies in special education: a comprehensive review and meta-analysis. Except. Child. 84, 7–26. doi: 10.1177/0014402917698008

[ref9] Brooks G. W. (2007). Teachers as readers and writers and as teachers of reading and writing. J. Educ. Res. 100, 177–191. doi: 10.3200/JOER.100.3.177-191

[ref10] Burroughs N. Gardner J. Lee Y. Guo S. Touitou I. Jansen K.. (2019). A review of the literature on teacher effectiveness and student outcomes. Teaching for excellence and equity: analyzing teacher characteristics, behaviors and student outcomes with TIMSS 6. doi: 10.1007/978-3-030-16151-4_2,

[ref11] Caprara G. V. Barbaranelli C. Steca P. Malone P. S. (2006). Teachers' self-efficacy beliefs as determinants of job satisfaction and students' academic achievement: a study at the school level. J. Sch. Psychol. 44, 473–490. doi: 10.1016/j.jsp.2006.09.001

[ref12] Cremin T. Baker S. (2010). Exploring teacher-writer identities in the classroom: conceptualising the struggle. Engl. Teach. Pract. Crit. 9, 8–25.

[ref13] Cremin T. Oliver L. (2017). Teachers as writers: a systematic review. Res. Pap. Educ. 32, 269–295. doi: 10.1080/02671522.2016.1187664

[ref14] Darling-Hammond L. Hyler M. E. Gardner M. (2017). Effective teacher professional development Palo Alto, CA: Learning Policy Institute.

[ref15] Darling-Hammond L. Richardson N. (2009). Research review/teacher learning: what matters. Educ. Leadersh. 66, 46–53.

[ref16] De Silva R. Graham S. (2015). The effects of strategy instruction on writing strategy use for students of different proficiency levels. System 53, 47–59. doi: 10.1016/j.system.2015.06.009

[ref17] De Smedt F. van Keer H. Merchie E. (2016). Student, teacher, and class-level correlates of Flemish late elementary school children’s writing performance. Read. Writ. Interdisc. J. 29, 833–868. doi: 10.1007/s11145-015-9590-z

[ref18] Desimone L. M. (2009). Improving impact studies of teachers’ professional development: toward better conceptualizations and measures. Educ. Res. 38, 181–199. doi: 10.3102/0013189X08331140

[ref19] Dostal H. Bowers L. Wolbers K. Gabriel R. (2015). “We are authors”: a qualitative analysis of deaf students’ writing during one year of strategic and interactive writing instruction (SIWI). Rev. Disabil. Stud. Int. J. 11, 1–19.

[ref20] Dostal H. M. Wolbers K. A. (2014). Developing language and writing skills of deaf and hard of hearing students: a simultaneous approach. Liter. Res. Instruct. 53, 245–268. doi: 10.1080/19388071.2014.907382

[ref21] Dostal H. Wolbers K. (2016). Examining student writing proficiencies across genres: results of an intervention study. Deaf. Educ. Int. 18, 159–169. doi: 10.1080/14643154.2016.1230415

[ref22] Dostal H. Wolbers K. Kilpatrick J. (2019). The language zone: differentiating writing instruction for students who are d/deaf and hard of hearing. Writ. Pedag. 11, 1–22. doi: 10.1558/wap.30045

[ref23] Draper M. C. Barksdale-Ladd M. A. Radencich M. C. (2000). Reading and writing habits of preservice teachers. Read. Horiz. J. Liter. Lang. Arts 40:3.

[ref24] Ekholm E. Zumbrunn S. DeBusk-Lane M. (2018). Clarifying an elusive construct: a systematic review of writing attitudes. Educ. Psychol. Rev. 30, 827–856. doi: 10.1007/s10648-017-9423-5

[ref25] Fauth B. Decristan J. Decker A.-T. Büttner G. Hardy I. Klieme E.. (2019). The effects of teacher competence on student outcomes in elementary science education: the mediating role of teaching quality. Teach. Teach. Educ. 86:102882. doi: 10.1016/j.tate.2019.102882

[ref26] Ferretti R. P. Lewis W. E. Andrews-Weckerly S. (2009). Do goals affect the structure of students’ argumentative writing strategies? J. Educ. Psychol. 101, 577–589. doi: 10.1037/a0014702

[ref27] Garberoglio C. L. Gobble M. E. Cawthon S. W. (2012). A national perspective on teachers’ efficacy beliefs in deaf education. J. Deaf. Stud. Deaf. Educ. 17, 367–383. doi: 10.1093/deafed/ens014, PMID: 22470179

[ref28] Garet M. S. Porter A. C. Desimone L. Birman B. F. Yoon K. S. (2001). What makes professional development effective? Results from a national sample of teachers. Am. Educ. Res. J. 38, 915–945. doi: 10.3102/00028312038004915

[ref29] Graham S. (2018). A revised writer(s)-within-community model of writing. Educ. Psychol. 53, 258–279. doi: 10.1080/00461520.2018.1481406

[ref30] Graham S. (2019). Changing how writing is taught. Rev. Res. Educ. 43, 277–303. doi: 10.3102/0091732X18821125

[ref31] Graham S. (2023). “Writer(s)-within-community model of writing as a lens for studying the teaching of writing” in The Routledge Handbook of International Research on Writing. ed. Horrowitz R. (New York: Routledge)

[ref32] Graham S. Bollinger A. Booth Olson C. D’Aoust C. MacArthur C. McCutchen D.. (2012). Teaching elementary school students to be effective writers: a practice guide (NCEE 20124058). Washington, DC: National Center for Education Evaluation and Regional Assistance, Institute of Education Sciences, U.S. Department of Education.

[ref33] Graham S. Collins A. A. Ciullo S. (2022). Special and general education teachers’ beliefs about writing and writing instruction. J. Learn. Disabil. 56, 163–179. doi: 10.1177/00222194221092156, PMID: 35502825

[ref01] Graham S. Collins A. A. Ciullo S. (2023). Special and general education teachers’ beliefs about writing and writing instruction. J. Learn. Disabil. 56, 163–179. doi: 10.1177/00222194221092156, PMID: 35502825

[ref34] Graham S. Harris K. R. (2002). “The road less traveled: prevention and intervention in written language” in Spelling, reading, and writing in children with language learning disabilities. eds. Butler K. Silliman E. (Mahwah: Erlbaum)

[ref35] Graham S. Harris K. Fink B. MacArthur C. A. (2001). Teacher efficacy in writing: a construct validation with primary grade teachers. Sci. Stud. Read. 5, 177–202. doi: 10.1207/S1532799Xssr0502_3

[ref36] Graham S. Perin D. (2007). Writing next: Effective strategies to improve writing of adolescents in middle and high schools – A report to Carnegie Corporation of new York. Washington, DC: Alliance for Excellent Education.

[ref37] Graham S. Wolbers K. Dostal H. Holcomb L. (2021). Does teacher efficacy predict writing practices of teachers of deaf and hard of hearing students. J. Deaf. Stud. Deaf. Educ. 26, 438–450. doi: 10.1093/deafed/enab012, PMID: 34017980

[ref38] Hall G. E. (1974). The concerns-based adoption model: a developmental conceptualization of the adoption process within educational institutions. Available at: https://files.eric.ed.gov/fulltext/ED111791.pdf (Accessed June 21, 2023).

[ref39] Hall G. E. (2013). Evaluating change processes: assessing extent of implementation (constructs, methods and implications). J. Educ. Adm. 51, 264–289. doi: 10.1108/09578231311311474

[ref40] Hall G. Dirksen D. George A. (2006). Measuring implementation in schools: levels of use. Austin: Southwest Educational Development Laboratory.

[ref41] Hall G. E. Hord S. M. (2020). Implementing change: Patterns, principles, and potholes (5th). Noida: Pearson.

[ref42] Hedges L. V. (2007). Effect sizes in cluster-randomized designs. J. Educ. Behav. Stat. 32, 341–370. doi: 10.3102/1076998606298043

[ref43] Hsiang T. P. Graham S. (2016). Teaching writing in grades 4–6 in urban schools in the greater China region. Read. Writ. 29, 869–902. doi: 10.1007/s11145-015-9597-5

[ref44] Hsiang T. P. Graham S. Wong P. M. (2018). Teaching writing in grades 7–9 in urban schools in Chinese societies in Asia. Read. Res. Q. 53, 473–507. doi: 10.1002/rrq.213

[ref45] Hsiang T. P. Graham S. Yang Y. M. (2020). Teachers’ practices and beliefs about teaching writing: a comprehensive survey of grades 1 to 3 teachers. Read. Writ. 33, 2511–2548. doi: 10.1007/s11145-020-10050-4

[ref46] Ingvarson L. Meiers M. Beavis A. (2005). Factors affecting the impact of professional development programs on teachers' knowledge, practice, student outcomes & efficacy. Educ. Policy Anal. Arch. 13:10. doi: 10.14507/epaa.v13n10.2005

[ref47] Kennedy M. M. (2016). How does professional development improve teaching? Rev. Educ. Res. 86, 945–980. doi: 10.3102/0034654315626800

[ref48] Lauermann F. ten Hagen I. (2021). Do teachers’ perceived teaching competence and self-efficacy affect students’ academic outcomes? A closer look at student-reported classroom processes and outcomes. Educ. Psychol. 56, 265–282. doi: 10.1080/00461520.2021.1991355

[ref49] Leko M. M. Brownell M. T. Sindelar P. T. Kiely M. T. (2015). Envisioning the future of special education personnel preparation in a standards-based era. Except. Child. 82, 25–43. doi: 10.1177/0014402915598782

[ref50] Littell R. D. Milliken G. A. Stroup W. W. Wolfinger R. D. Schabenberger O. (2006). SAS for mixed models (2nd). North California: SAS Institute.

[ref51] Mayer C. Trezek B. J. (2015). Early literacy development in deaf children. Oxford:Oxford University Press.

[ref52] McKinney M. Giorgis C. (2009). Narrating and performing identity: literacy specialists' writing identities. J. Lit. Res. 41, 104–149. doi: 10.1080/10862960802637604

[ref53] McMaster K. L. Lembke E. S. Shin J. Poch A. L. Smith R. A. Jung P.-G.. (2020). Supporting teachers’ use of data-based instruction to improve students’ early writing skills. J. Educ. Psychol. 112, 1–21. doi: 10.1037/edu0000358

[ref54] Piasta S. B. Connor C. M. Fishman B. J. Morrison F. J. (2009). Teacher’s knowledge of literacy concepts, classroom practices, and student reading growth. Sci. Stud. Read. 13, 224–248. doi: 10.1080/10888430902851364

[ref55] Rietdijk S. van Weijen D. Jassen T. van den Bergh H. Rijlaarsdam G. (2018). Teaching writing in primary education: classroom practice, time, teachers’ beliefs and skills. J. Educ. Psychol. 110, 640–663. doi: 10.1037/edu0000237

[ref56] Rogers L. A. Graham S. (2008). A meta-analysis of single subject design writing intervention research. J. Educ. Psychol. 100, 879–906. doi: 10.1037/0022-0663.100.4.879

[ref57] Saddler B. Graham S. (2005). The effects of peer-assisted sentence-combining instruction on the writing performance of more and less skilled young writers. J. Educ. Psychol. 97, 43–54. doi: 10.1037/0022-0663.97.1.43

[ref58] Salgado R. Mundy M. A. Kupczynski L. Challoo L. (2018). Effects of teacher efficacy, certification route, content hours, experiences and class size on student achievement. J. Instruct. Pedag. 21, 1–18.

[ref59] Strassman B. K. Schirmer B. (2013). Teaching writing to deaf students: does research offer evidence for practice? Remedial Spec. Educ. 34, 166–179. doi: 10.1177/0741932512452013

[ref60] Street C. (2003). Pre-service teachers' attitudes about writing and learning to teach writing: implications for teacher educators. Teach. Educ. Q. 30, 33–50.

[ref61] Thames M. H. Ball D. L. (2010). What math knowledge does teaching require? Teach. Child. Math. 17, 220–229. doi: 10.5951/TCM.17.4.0220

[ref62] Tracy B. Reid R. Graham S. (2009). Teaching young students strategies for planning and drafting stories: the impact of self-regulated strategy development. J. Educ. Res. 102, 323–332. doi: 10.3200/JOER.102.5.323-332

[ref63] Troia G. A. Graham S. (2002). The effectiveness of a highly explicit, teacher-directed strategy instruction routine: changing the writing performance of students with learning disabilities. J. Learn. Disabil. 35, 290–305. doi: 10.1177/00222194020350040101, PMID: 15493239

[ref64] Tschannen-Moran M. Woolfolk Hoy A. Hoy W. K. (1998). Teacher efficacy: its meaning and measure. Rev. Educ. Res. 68, 202–248. doi: 10.3102/00346543068002202

[ref65] Van Driel J. H. Berry A. (2012). Teacher professional development focusing on pedagogical content knowledge. Educ. Res. 41, 26–28. doi: 10.3102/0013189X11431010

[ref66] Watkins J. Peterson A. Metah J. (2018). Transforming school districts to support deeper learning for all: a hypothesis. Available at: https://www.deeperlearningdozen.org/ (Accessed April 29, 2023).

[ref67] Whitney A. E. (2009). Writer, teacher, person: tensions between personal and professional writing in a National Writing Project summer institute. Engl. Educ. 41, 235–258.

[ref68] Whyte A. Lazarte A. Thompson I. Ellis N. Muse A. Talbot R. (2007). The National Writing Project, teachers' writing lives, and student achievement in writing. Action Teach. Educ. 29, 5–16. doi: 10.1080/01626620.2007.10463444

[ref69] Wijekumar K. Beerwinkle A. L. Harris K. R. Graham S. (2019). Etiology of teacher knowledge and instructional skills for literacy at the upper elementary grades. Ann. Dyslexia 69, 5–20. doi: 10.1007/s11881-018-00170-6, PMID: 30607812

[ref70] Wilson S. D. (2013). Caring leadership applied in the classroom to embrace the needs of students. J. Coll. Teach. Learn. 10, 23–28. doi: 10.19030/tlc.v10i1.7527

[ref71] Wolbers K. A. (2008a). Strategic and interactive writing instruction (SIWI) apprenticing deaf students in the construction of English text. Int. J. Appl. Linguis. 156, 299–326. doi: 10.2143/ITL.156.0.2034441

[ref72] Wolbers K. A. (2008b). Using balanced and interactive writing instruction to improve the higher order and lower order writing skills of deaf students. J. Deaf. Stud. Deaf. Educ. 13, 257–277. doi: 10.1093/deafed/enm052, PMID: 17965129

[ref73] Wolbers K. A. Dostal H. M. Bowers L. M. (2012). “I was born full deaf.” written language outcomes after 1 year of strategic and interactive writing instruction. J. Deaf. Stud. Deaf. Educ. 17, 19–38. doi: 10.1093/deafed/enr018 21571902

[ref74] Wolbers K. Dostal H. Cihak D. Holcomb L. (2020). Written language outcomes of deaf elementary students engaged in authentic writing. J. Deaf. Stud. Deaf. Educ. 25, 224–238. doi: 10.1093/deafed/enz047, PMID: 32034407

[ref75] Wolbers K. Dostal H. Graham S. Branum-Martin L. Holcomb L. (2022). Specialized writing instruction for deaf students: a randomized controlled trial. Except. Child. 88, 185–204. doi: 10.1177/00144029211050849

[ref76] Wolbers K. Dostal H. Graham S. Branum-Martin L. Kilpatrick J. Saulsburry R. (2018). Strategic and interactive writing instruction: an efficacy study in grades 3-5. J. Educ. Dev. Psychol. 8, 99–117. doi: 10.5539/jedp.v8n1p99

[ref77] Wolbers K. Dostal H. Graham S. Cihak D. Kilpatrick J. Saulsburry R. (2015). The writing performance of elementary students receiving strategic and interactive writing instruction. J. Deaf. Stud. Deaf. Educ. 20, 385–398. doi: 10.1093/deafed/env022, PMID: 26101210

[ref78] Wolbers K. Dostal H. Holcomb L. (2023). Teacher reports of secondary writing instruction with deaf students. Advanced online publication. J. Lit. Res. 55, 28–50. doi: 10.1177/1086296X231163124

[ref79] Wolbers K. Dostal H. Skerrit P. Stephenson B. (2016). A three-year study of a professional development program's impact on teacher knowledge and classroom implementation of strategic and interactive writing instruction. J. Educ. Res. 110, 61–71. doi: 10.1080/00220671.2015.1039112

[ref80] Yarrow F. Topping K. J. (2001). Collaborative writing: the effects of metacognitive prompting and structured peer interaction. Br. J. Educ. Psychol. 71, 261–282. doi: 10.1348/000709901158514, PMID: 11449936

[ref81] Zee M. Koomen H. M. Y. (2016). Teacher self-efficacy and its effects on classroom processes, student academic adjustment, and teacher well-being: a synthesis of 40 years of research. Rev. Educ. Res. 86, 981–1015. doi: 10.3102/0034654315626801

