# Supplementary material for: Writing knowledge, practices, efficacy, interests, attitudes, and beliefs of deaf education teachers: a randomized controlled trial
Source: Front Psychol. 2023 Jul 7;14:1214246. doi: 10.3389/fpsyg.2023.1214246 (PMC10361062; doi:10.3389/fpsyg.2023.1214246)
Supplement: Supplementary file 1 [file Table_1.DOCX]

Table S1. Correlation matrix of main outcomes.

| Time | Variable | Know | Teach | Support | Efficacy | Know | Teach | Support | Efficacy |
| --- | --- | --- | --- | --- | --- | --- | --- | --- | --- |
| Pretest | Knowledge | 1.00 | -0.11 | -0.15 | -0.10 | 0.24 | -0.03 | -0.06 | 0.00 |
|  | Teaching Writing | -0.11 | 1.00 | 0.72 | 0.38 | -0.33 | 0.09 | 0.15 | 0.19 |
|  | Supporting Writing | -0.15 | 0.72 | 1.00 | 0.23 | -0.26 | 0.12 | 0.30 | 0.09 |
|  | Efficacy | -0.10 | 0.38 | 0.23 | 1.00 | -0.07 | 0.03 | 0.09 | 0.50 |
| Posttest | Knowledge | 0.24 | -0.33 | -0.26 | -0.07 | 1.00 | 0.45 | 0.34 | 0.40 |
|  | Teaching Writing | -0.03 | 0.09 | 0.12 | 0.03 | 0.45 | 1.00 | 0.84 | 0.38 |
|  | Supporting Writing | -0.06 | 0.15 | 0.30 | 0.09 | 0.34 | 0.84 | 1.00 | 0.52 |
|  | Efficacy | 0.00 | 0.19 | 0.09 | 0.50 | 0.40 | 0.38 | 0.52 | 1.00 |
|  | MEAN | 0.16 | 4.04 | 4.51 | 4.41 | 2.03 | 4.55 | 4.67 | 4.52 |
|  | STD | 0.42 | 1.10 | 0.88 | 0.69 | 1.92 | 1.16 | 0.97 | 0.67 |

*Note*. Pretest n = 50. Posttest n = 48. Shading below diagonal indicates within-construct stability over time.

Table S2. Correlation matrix of exploratory outcomes.

| Time | Variable | Interest | Attitude | Effort | Innate | Expert | Certain | Interest | Attitude | Effort | Innate | Expert | Certain |
| --- | --- | --- | --- | --- | --- | --- | --- | --- | --- | --- | --- | --- | --- |
| Pre | Interest | 1.00 | 0.41 | 0.12 | -0.18 | 0.06 | 0.11 | 0.53 | 0.31 | 0.10 | -0.15 | -0.14 | 0.05 |
|  | Attitude | 0.41 | 1.00 | 0.26 | -0.14 | 0.20 | -0.05 | 0.33 | 0.83 | 0.11 | -0.10 | 0.17 | -0.12 |
|  | Effort | 0.12 | 0.26 | 1.00 | 0.10 | 0.54 | 0.08 | 0.03 | 0.22 | 0.63 | -0.06 | 0.44 | -0.09 |
|  | Innate | -0.18 | -0.14 | 0.10 | 1.00 | 0.42 | 0.50 | 0.04 | -0.09 | 0.01 | 0.66 | 0.18 | 0.27 |
|  | Expert | 0.06 | 0.20 | 0.54 | 0.42 | 1.00 | 0.37 | -0.02 | 0.17 | 0.14 | 0.18 | 0.44 | 0.00 |
|  | Certain | 0.11 | -0.05 | 0.08 | 0.50 | 0.37 | 1.00 | -0.11 | 0.09 | -0.07 | 0.20 | -0.02 | 0.48 |
| Post | Interest | 0.53 | 0.33 | 0.03 | 0.04 | -0.02 | -0.11 | 1.00 | 0.31 | -0.04 | 0.01 | -0.08 | 0.07 |
|  | Attitude | 0.31 | 0.83 | 0.22 | -0.09 | 0.17 | 0.09 | 0.31 | 1.00 | 0.11 | 0.04 | 0.22 | 0.00 |
|  | Effort | 0.10 | 0.11 | 0.63 | 0.01 | 0.14 | -0.07 | -0.04 | 0.11 | 1.00 | 0.15 | 0.41 | 0.08 |
|  | Innate | -0.15 | -0.10 | -0.06 | 0.66 | 0.18 | 0.20 | 0.01 | 0.04 | 0.15 | 1.00 | 0.35 | 0.22 |
|  | Expert | -0.14 | 0.17 | 0.44 | 0.18 | 0.44 | -0.02 | -0.08 | 0.22 | 0.41 | 0.35 | 1.00 | 0.13 |
|  | Certain | 0.05 | -0.12 | -0.09 | 0.27 | 0.00 | 0.48 | 0.07 | 0.00 | 0.08 | 0.22 | 0.13 | 1.00 |
|  | MEAN | 4.52 | 4.41 | 4.59 | 2.18 | 3.53 | 2.91 | 4.85 | 4.37 | 4.72 | 2.28 | 3.50 | 2.83 |
|  | STD | 1.05 | 0.95 | 0.74 | 0.72 | 0.66 | 0.59 | 1.05 | 0.97 | 0.68 | 0.65 | 0.79 | 0.61 |
|  | n | 50 | 49 | 50 | 50 | 50 | 50 | 48 | 47 | 48 | 48 | 48 | 48 |

*Note*. Shading below the diagonal indicates within-construct stability over time.
